# A Comparative Study of Five Target Volume Definitions for Radiotherapy in Glioblastoma Multiforme

**DOI:** 10.3390/medicina61101860

**Published:** 2025-10-16

**Authors:** Kamuran Ibis, Kubra Ozkaya Toraman, Canan Koksal Akbas, Ozlem Guler Guniken, Korhan Kokce, Sezi Ceren Gunay, Rasim Meral, Musa Altun

**Affiliations:** 1 Department of Radiation Oncology, Institute of Oncology, Istanbul University, 34093 Istanbul, Türkiye; kubra.ozkaya@istanbul.edu.tr (K.O.T.); ozlem.guler@istanbul.edu.tr (O.G.G.); korhan.kokce@istanbul.edu.tr (K.K.); sezi.gunay@istanbul.edu.tr (S.C.G.); rasim.meral@istanbul.edu.tr (R.M.); musa.altun@istanbul.edu.tr (M.A.); 2 Department of Medical Physics, Institute of Oncology, Istanbul University, 34093 Istanbul, Türkiye; canan.koksal@istanbul.edu.tr

**Keywords:** glioblastoma multiforme, radiotherapy, volume delineation

## Abstract

*Background and Objectives:* This study aimed to compare target volumes and organ-at-risk (OAR) doses using five different volume definitions in radiotherapy (RT) planning of patients with glioblastoma multiforme (GBM). *Materials and Methods:* Rigid image fusion was performed using simulation computed tomography and postoperative magnetic resonance imaging scans of 20 patients with GBM. Volumetric modulated arc therapy (VMAT) plans were generated according to three two-phase protocols—American Brain Tumor Consortium (ABTC), North Central Cancer Treatment Group/Alliance (NCCTG/Alliance), and Radiation Therapy Oncology Group/NRG (RTOG/NRG)—and two single-phase protocols—European Organisation for Research and Treatment of Cancer (EORTC) and European Society for Radiotherapy and Oncology–European Association of Neuro-Oncology (ESTRO/EANO)—each delivering a total dose of 60 Gy. OARs and dose constraints were evaluated. Statistical analysis was performed using the paired sample *t*-test. *Results:* The ESTRO/EANO volume had the smallest median PTV overall (*p* < 0.001). The lowest brain-PTV Dmean in the initial phase was observed in the ABTC group, followed closely by ESTRO/EANO (*p* < 0.001). Among boost volumes, the ABTC volume was the smallest, and the median brain-PTV Dmean was lowest in the ESTRO/EANO volume. ESTRO/EANO provided the lowest doses for contralateral and ipsilateral cochlea Dmean, brainstem D1cc, and contralateral lens Dmax. Notably, both EORTC and ESTRO/EANO plans maintained OAR doses within acceptable constraints, with ESTRO/EANO achieving the most consistently minimised exposure. *Conclusions:* Reduced irradiated brain volume, acceptable OAR preservation and practical applicability, the use of ESTRO-EANO and EORTC target volumes in radiotherapy of glioblastoma multiforme may provide dosimetric advantages that require further validation in clinical outcome studies.

## 1. Introduction

Glioblastoma Multiforme (GBM) is the most common primary malignant brain tumor in adults and remains one of the most aggressive and fatal human cancers [1]. Despite advances in treatment, five-year survival rates remain exceedingly low [2]. Since 2005, the standard of care has consisted of maximal safe surgical resection followed by concurrent chemoradiotherapy and adjuvant temozolomide [3].

Over the decades, radiotherapy (RT) target volumes for high-grade glial tumors have progressively decreased in size [4]. Reducing irradiated brain volume is essential, as larger treatment fields are associated with increased risk of neurotoxic effects, including radiation necrosis and cognitive impairment [5,6].

Currently, different cooperative groups have proposed varied volume definitions, primarily using postoperative magnetic resonance imaging (MRI). In 2016, the American Society for Radiation Oncology (ASTRO) summarized the target volume definitions used in four major GBM clinical trials [7]. North American radiation oncology cooperative groups, including the American Brain Tumor Consortium (ABTC), the North Central Cancer Treatment Group/Alliance (NCCTG/Alliance), and the Radiation Therapy Oncology Group/NRG (RTOG/NRG), generally use a two-phase approach. The initial phase targets the peritumoral edema (as seen on T2/FLAIR MRI) along with the resection cavity and any residual contrast-enhancing tumor (on T1-weighted imaging), followed by a boost phase focused solely on the cavity and residual tumor [7]. Although similar in structure, these protocols vary in terms of clinical target volume (CTV) margins and prescribed doses. The rationale for including edema in RT planning is based on the belief that these areas contain infiltrative tumor cells [8,9,10].

In 2023, the European Society for Radiotherapy and Oncology/European Association of Neuro-Oncology (ESTRO/EANO) released updated consensus guidelines recommending a single CTV encompassing the resection cavity and residual T1-enhancing regions with a 15 mm isotropic margin. FLAIR abnormalities suspected to represent non-enhancing tumors should also be included [11]. In this study, we aimed to dosimetrically compare the planning target volumes and organ-at-risk (OAR) doses in volumetric modulated arc therapy (VMAT) treatment plans created using 5 different target volume definitions in radiotherapy for GBM.

## 2. Materials and Methods

This dosimetric study included 20 patients previously diagnosed with GBM who had received RT and concurrent temozolomide therapy at our clinic. All patients underwent simulation using thermoplastic masks for head immobilization. Simulated computed tomography (CT) scans were obtained with a 3 mm slice thickness using a Philips Brillance Big Bore 4D CT device (Philips Medical Systems, Best, The Netherlands) for treatment planning. Postoperative cranial MRI images including contrast-enhanced T1-weighted and T2/FLAIR sequences were obtained, and rigid fusion was performed with simulated CT using Mim Software Version 6.5 (MIM Software Inc., Cleveland, OH, USA). Image registration is important for determining the treatment target volume. Performing an MRI in the treatment position with an immobilization mask can reduce uncertainties associated with image registration, but it is often not easily achieved [11]. Registration can be interactive, semi-automatic, or fully automatic. Manual adjustment of the registration is recommended following automatic registration. The role of user in image registration is important. Rigid fusion allows translation in three directions and rotations around three axes. The maximum dimensionality of the transformation is six dimensions. As reported in AAPM Radiation Therapy Committee Task Force Report Group No. 132, uncertainties can be reduced by using the orbits, optic nerves, brainstem, ventricles, Sella Turcica, nose, external auditory canal (may be useful for minimizing rotation/roll), and clivus (for minimizing rotation/yaw errors) sagittal suture as anatomical landmark mappings [12]. Rigid image registration was performed taking into account the criteria of TAG 132.

Then, target volumes and prescribed doses were determined according to five different GBM target volume definitions derived from clinical studies and international guidelines: three two-phase ABTC, NCCTG/Alliance, RTOG/NRG, and two single-phase EORTC, ESTRO/EANO. Detailed volume definitions are summarized in Table 1, based on the 2016 ASTRO guideline and the ESTRO/EANO guideline [7,11].

Organ at risk and dose constraints were determined based on the ESTRO-ACROP guideline and the study by Scoccianti et al. [13,14]. The ESTRO/EANO guidelines report that critical OARs, which should be identified as a minimum requirement, include the optic nerves, optic chiasm, eyes, lenses, brain, and brainstem, which should be considered during the planning process. It is emphasized that all of these organs may compromise PTV coverage. Non-critical OARs include the cochleas, lacrimal glands, pituitary gland, hypothalamus, and hippocampus. For these structures, dose constraints can be used as a guide during planning optimization, but PTV restriction is not explicitly recommended unless critical dose constraints, such as those to the brainstem or optic tract, cannot otherwise be met. For hippocampal sparing, neurocognitive data to support its use when planning radiotherapy for glioblastoma patients are currently lacking [11]. Defined OARs were the optic nerves, optic chiasm, eyes, retinae, lenses, brainstem, cochleae, lacrimal glands, and pituitary gland. Volumes were created taking into account anatomical barriers.

Radiotherapy planning was conducted using the Eclipse Treatment Planning System (Varian Medical Systems, Palo Alto, CA, USA). VMAT plans were generated using three full arcs with different collimator angles, with a total dose of 60 Gy in 30 fractions. Dose calculations were performed using the Anisotropic Analytical Algorithm (AAA). To ensure comparability, identical optimization parameters were used for all plans. The quality of the RT was evaluated by two indices calculated according to these two formulae: Homogeneity Index (HI): (D2% − D98%)/D50% and Conformity Index (CI): Vri/PTV volume [15].

A comparison of absolute volumes of all PTVs and brain-PTV mean dose (Dmean) across all treatment plans was planned. First, the initial PTVs in two-phase treatment plans, the PTVs in single-phase treatment plans (a total of five PTVs), and the brain-PTV mean doses obtained from these plans were compared. Next, the boost volumes in 2-phase treatment plans, the PTVs in single-phase treatment plans (a total of five PTVs), and the brain-PTV Dmean obtained from the total dose of five different plans were compared. OAR doses were compared in plans that applied 60 Gy in 30 fractions using VMAT according to five different target volume definitions. Dose constraints and detailed OAR definitions are listed in Table 2.

The paired-samples *t*-test was used for compared target volumes and OARs doses. Statistical analysis was performed using SPSS for Windows version 22 software (SPSS Inc., Chicago, IL, USA). A *p*-value of <0.05 considered statistically significant. For pairwise comparisons, Bonferroni correction was applied, and *p* value less than 0.005 was considered significant, since 10 pairwise comparisons needed to compare *n* = 5 dependent groups. This study was approved by the Clinical Research Ethics Committee of Istanbul Faculty of Medicine, Istanbul University (Approval Number: 2025/197, Date: 21 February 2025).

## 3. Results

Among the 20 patients, tumor locations were as follows: right frontotemporal (*n* = 1), right occipitoparietal (*n* = 1), right parietal (*n* = 1), right temporoparietal (*n* = 1), right temporal (*n* = 5), right temporo-occipital (*n* = 1), left frontal (*n* = 5), left parietal (*n* = 2), left parieto-occipital (*n* = 1), and left temporal (*n* = 1). Gross total resection was performed in 14 patients (70%), while six patients (30%) underwent subtotal resection.

**Figure 1:**
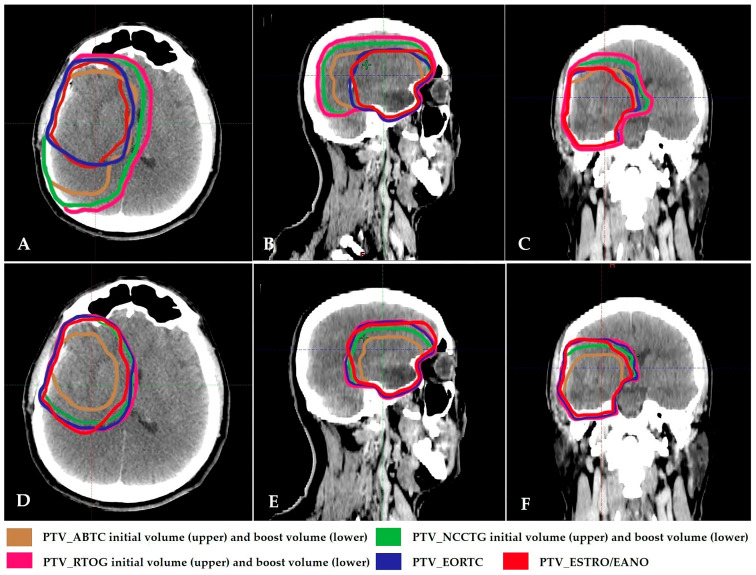
(**A**–**C**): Initial PTV contours of 2-phase GBM treatment volumes and PTV contours of single-phase EORTC and ESTRO treatment volumes in axial, sagittal, and coronal section simulation CT images. (**D**–**F**): Boost PTV contours of 2-phase GBM treatment volumes and PTV contours of single-phase EORTC and ESTRO treatment volumes in axial, sagittal, and coronal section simulation CT images. ABTC: American Brain Tumor Consortium; NCCTG/Alliance: North Central Cancer Treatment Group/Alliance; RTOG/NRG: Radiation Therapy Oncology Group/NRG; EORTC: European Organization for Research and Treatment of Cancer; ESTRO/EANO: European Society for Radiotherapy & Oncology/European Association of Neuro-Oncology.

When comparing the PTV initials in three 2-phase treatment plans, the PTVs in two single-phase treatment plans, and the brain-PTV Dmean obtained from these plans, the results are as follows. The median PTV volumes for the initial phase were 293.8 cm^3^ for ABTC, 436.4 cm^3^ for NCCTG/Alliance, and 519.7 cm^3^ for RTOG/NRG. For the two single-phase plans, the median PTV volumes were 299 cm^3^ for EORTC and 220.4 cm^3^ for ESTRO/EANO. When these five volumes were compared, all were statistically different from one another, except for ABTC initial and EORTC, which showed no statistically significant difference (ABTC initial vs. EORTC volume; *p* = 0.083; Table 3; Figure 1A–C). In terms of Brain-PTV Dmean, the lowest dose was observed in the ABTC plan, which differed significantly from the other four groups. Additionally, the ESTRO/EANO plan yielded a lower Brain-PTV Dmean compared to NCCTG/Alliance initial, RTOG/NRG initial, and EORTC volumes (Table 3). In all plans, HI was close to 0 and CI was close to 1. The resulting PTV volume, PTV Dmean doses, HI and CI values when comparing the PTV initials in the three 2-phase treatment plans and the PTVs in the two single-phase treatment plans are presented in Table S2 with details.

**Figure 2:**
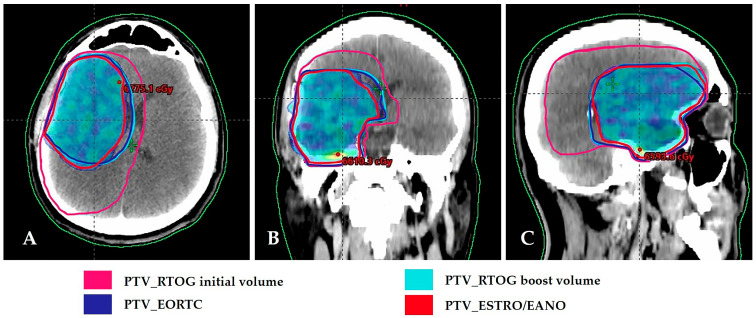
Comparison of RTOG, EORTC, and ESTRO/EANO planning target volumes. Dose distribution of 60 Gy in PTV of ESTRO/EANO. (**A**) Axial view; (**B**) Coronal view; (**C**) Sagittal view. RTOG/NRG: Radiation Therapy Oncology Group/NRG; EORTC: European Organization for Research and Treatment of Cancer; ESTRO/EANO: European Society for Radiotherapy & Oncology/European Association of Neuro-Oncology.

When the boost volumes in three 2-phase treatment plans, the PTVs in two single-phase treatment plans, and brain-PTV Dmean obtained from these plans were compared, the results were as follows. For the three boost volumes, median PTVs were 144.5 cm^3^ for ABTC, 233 cm^3^ for NCCTG/Alliance, and 292 cm^3^ for RTOG/NRG (Figure 1D–F). The two single-phase volumes, EORTC and ESTRO/EANO, were 299 cm^3^ and 220.4 cm^3^, respectively (Figure 2). In all plans, HI was close to 0 and CI was close to 1. The PTV volume, PTV Dmean doses, HI and CI values obtained when comparing PTV boosts in three 2-phase treatment plans and PTVs in two single-phase treatment plans are presented in Table S3 with details.

All five PTV volumes were statistically different from one another, except for RTOG/NRG boost and EORTC, between which no significant difference was found (*p* = 0.078; Table 4). When comparing Brain-PTV Dmean across the five plans, the lowest dose was again seen in the ESTRO/EANO plan. No statistically significant difference was observed between the ABTC boost volume and the EORTC volume (*p* = 0.481; Table 4).

All OAR doses were detailed in Table S1. The median brainstem maximum dose (Dmax) was the highest in the RTOG/NRG and the lowest in the ABTC. Brainstem maximum dose to 1 cc (D1cc), although within acceptable limits in all plans, differed significantly; the lowest value was in ESTRO/EANO (median: 44.9 Gy). Optic chiasm Dmax was lowest in the ABTC plan. While there was a significant difference among the groups, no statistical difference was observed between ABTC vs. EORTC and ABTC vs. ESTRO/EANO.

Contralateral cochlear Dmean was the lowest in ESTRO/EANO (median: 4.4 Gy). Ipsilateral cochlear Dmean was lowest in ESTRO/EANO (median: 17.7 Gy) and highest in RTOG/NRG (median: 24.6 Gy), without statistically significant difference between them.

Pituitary Dmax was lowest in ABTC and ESTRO/EANO plans (both median: 33.5 Gy) and highest in RTOG/NRG (median: 45.1 Gy). Both contralateral and ipsilateral eye Dmax were lowest in ABTC (median: 16 Gy and 19 Gy, respectively).

Contralateral lacrimal gland Dmax was lowest in ABTC (median: 14.9 Gy), followed by EORTC (median: 16.4 Gy) and ESTRO/EANO (median: 16.8 Gy), without significant difference (*p* = 0.044 and *p* = 0.019, respectively). Ipsilateral lacrimal gland Dmax was lowest in ABTC (median: 20.6 Gy), followed by EORTC (median: 23.4 Gy) and ESTRO/EANO (median: 25 Gy). The difference between ABTC and EORTC was not statistically significant (*p* = 0.047).

Contralateral lens Dmax was lowest in ESTRO/EANO (median: 5.2 Gy). No statistical difference was found between ABTC (5.9 Gy) and EORTC (*p* = 0.295), and ESTRO/EANO (*p* = 0.017). Ipsilateral lens Dmax was also lowest in ESTRO/EANO (median: 5.1 Gy). No significant difference was found between ABTC and EORTC (both 6.2 Gy; *p* = 0.101), but both were significantly lower than NCCTG/Alliance and RTOG/NRG.

Although statistically significant differences were detected in OAR doses according to different treatment volumes, all OARs were observed within the dose constraints expected for cochlea ipsilaterally. Dmean in one patient with ESTRO/EANO treatment planning of 48.2 Gy, eye Dmax ipsilateral in one patient with ABTC, NCCTG, RTOG/NRG, EORTC treatment planning of >45 Gy (46.4–49.4 Gy), lacrimal gland Dmax ipsilateral in one patient with NCCTG and RTOG treatment planning of >40 Gy (43.6, 44 Gy), lens Dmax ipsilateral in 3 patients with NCCTG, RTOG, and 3 patients with EORTC of >10 Gy.

A homogeneous dose distribution was achieved in VMAT plans based on both initial and boost volumes across all five treatment plannings. The CI for the three initial plans and the two single-phase plans was approximately 1, while the CI for the three two-phase plans was greater than 1, due to the sequential boost plans.

## 4. Discussion

In this dosimetric comparison study, we evaluated five widely recognized RT volume definitions to assess their impact on target volume size and dose exposure to critical OARs. When 3 PTV initial and 2 single-phase volumes were compared, all were statistically different from each other except ABTC initial and EORTC; no statistically significant difference was observed in ABTC initial and EORTC (*p* = 0.083). The lowest Brain-PTV Dmean doses were observed in the ABTC initial schedule, which was significantly different from the other four groups. Furthermore, the ESTRO/EANO schedule produced a lower Brain-PTV Dmean value compared to the other schedules. Our findings demonstrate that the ABTC, EORTC, and ESTRO/EANO protocols offer significant advantages in minimizing treatment volumes and OAR doses. When comparing brain-PTV Dmean doses, there is no difference between ABTC and EORTC volumes, while ESTRO/EANO has the lowest values. Although the ABTC target volume definition includes T2 edema in phase 1, the treatment volume is small because it recommends a 5 mm CTV margin in both phases 1 and 2. It is particularly noteworthy that the volume administered at 60 Gy is minimal compared to other target volume definitions.

As we write this article, an updated ASTRO guideline for RT treatment of Grade 4 adult diffuse gliomas, including GBM, was published in 2025 [16]. Therefore, we were unable to include these target volumes in our study. The new ASTRO guideline includes two recommendations for target volume for Grade 4 adult diffuse gliomas, including GBM. In the single-phase targeting approach, the GTV is generally considered the resection cavity and residual tumor detected on post-contrast T1-weighted MRI sequences. The CTV is considered a 10 to 20 mm extension from the GTV and is then adjusted to include abnormal FLAIR/T2-weighted imaging changes (non-enhancing tumor). In the 2-phase approach, GTV1 includes the resection cavity and residual enhancement, including postoperative T1 post-contrast MRI and T2/FLAIR changes (non-enhancing tumor), and cone-down GTV2 is limited to the resection cavity and residual enhancement on postoperative T1 post-contrast MRI. CTV1 and CTV2 include a 10 to 20 mm extension of the involved GTV, respectively [16].

The critical issue for defining targets is to minimize side effects without compromising disease control. Cranial irradiation may lead to brain atrophy and is associated with delayed complications such as cognitive deficits, progressive global dementia, apathy, and personality changes [6]. Consequently, careful identification and protection of intracranial OARs during GBM radiotherapy is crucial [14]. The development of radiation-induced toxicity is influenced by factors such as the irradiated volume, total dose, fraction size, and overall treatment duration [17,18,19].

Studies examining recurrence patterns in patients treated with RT using current technologies and concurrent and adjuvant temozolomide, with the target volume determined using MRI images, will be decisive in CTV margins. These studies generally consist of analyses of prospective phase II and retrospective studies. Of the eight studies examining recurrence patterns in GBM published between 2009 and 2014, four used a single-phase [20,21,22,23] treatment method, while the others [24,25,26,27] used a two-phase treatment method. CTV margins ranged from 5 to 30 mm. In all studies, 80% or more of the recurrences were central or in-field.

When more recent studies are evaluated; Guram et al. analyzed the treatment plans and outcomes of 276 patients with high-grade gliomas (*n* = 191), 71.5% of whom had GBM, who were treated with temozolomide and RT after surgical resection. The 61.2 Gy dose is split into an initial 45 Gy dose in 25 fractions, followed by a 16.2 Gy boost in 9 fractions. In the initial phase, they added the resection cavity, T1 contrast-enhanced area, and edema in T2 or FLAIR to create PTV1 with a margin of 0.4 cm or 1 cm. The cone-down volume encompasses the enhancing tumor volume and resection cavity. This volume is also expanded by 0.4 or 1 cm to create PTV2. Large (1 cm) margin GBM patients accounted for 55.4% (*n* = 148) of the total patients, while small margin (0.4 cm) GBM patients accounted for 16.1% (*n* = 43) of the total patients. Treatment margin did not affect the outcome. The 1 cm margin subgroup (*n* = 212) showed median PFS and OS times of 10.7 and 19.1 months, respectively, and the 0.4 cm margin subgroup (*n* = 55) had times of 10.2 and 19.3 months. They reported no significant differences in outcomes when compared with historical standard treatments using 2–3 cm margins. The details of recurrence patterns were not reported in this study [28]. The volumes in this study are similar to the target volume definition of ABTC.

Kumar et al. conducted a phase 2 randomized trial involving 50 patients to investigate the effect of radiation volume on survival and quality of life in glioblastoma, comparing the RTOG and The University of Texas MD Anderson Cancer Centre (MDACC) protocols. Patients in both arms were treated with 3D-CRT in 30 fractions to a total of 60 Gy in two phases. However, unlike the RTOG protocol, a 2 cm isotropic margin was used in the initial phase without including peritumoral edema in the target volume. In the boost phase, a 2 cm margin was used in the RTOG protocol, while a 0.5 cm margin was used in the MDACC protocol. When recurrence patterns were evaluated, central recurrence was 12 (75%) and 11 (68.75%), in-field recurrence was 2 (12.5%) and 3 (18.5%), marginal recurrence was 2 (12.5%) and 1 (6.25%), distant recurrence was 0 and 1 (6.25%) in the RTOG and MDACC protocols, respectively, and no statistical difference was found (*p* = 0.81). Dosimetric analysis revealed significantly lower boost-phase planning treatment volumes and V60 Gy in the MDACC arm (*p* = 0.001 and 0.013, respectively). No significant differences were observed in doses concerning organs at risk, acute toxicity, or recurrence patterns (*p* > 0.05). QoL of patients was significantly better in the MDACC group in all domains except cognitive (*p* < 0.05) [29]. In the MDACC protocol, the volume defined in the initial phase is similar to the EORTC volume definition, while the boost phase volume is similar to the ABTC boost phase volume. Slight differences in the applied doses were observed (40 Gy in 20 fractions in the initial phase and 20 Gy in 10 fractions in the boost phase).

Tu et al. included 68 patients with GBM who underwent temozolomide and RT and subsequently developed recurrence in their study. The patients were irradiated according to MDACC guidelines. Recurrences were observed in 47 (69.1%) patients locally, in 12 (17.7%) patients distantly, and in 9 (13.2%) patients in both locations. When T1-enhanced sequences were analyzed, all local recurrences occurred within 2 cm and 94.8% (55/58) within 1 cm of the original GTV; When T2-FLAIR sequences were analyzed, all local recurrences were within 1.5 cm, and 98.3% (57/58) occurred within 0.5 cm of the original GTV [30]. When recurrence patterns were analyzed, the ESTRO/EANO target volume definition appears to be a suitable option for patients to be treated in a single phase.

Zheng et al. reported in a retrospective analysis of HGG patients treated before 2014 that excluding T2/FLAIR hyperintensity from the target volume did not increase marginal recurrence [31,32]. They defined the GTV as the enhanced area and resection cavity on contrast-enhanced T1-weighted MRI sequences. When creating CTV1 and CTV2, they added margins of 1 and 2 cm to the GTV, respectively. They defined a dose of 54 Gy in 30 fractions for PTV2 and 60 Gy in 30 fractions for PTV1, and administered RT using intensity-modulated radiotherapy (IMRT) with the simultaneous integrated boost technique. All patients received concurrent RT and temozolomide chemotherapy. A total of 162 GBM cases were treated, and the clinical data and MRI images of 55 patients with recurrent GBM were retrospectively analyzed. The median OS and PFS were 17.7 and 7.0 months, respectively. Among the patients, 44 (80%) had central recurrence, 2 (3.6%) had in-field recurrence, 1 (1.8%) had marginal recurrence, 11 (20%) had distant recurrence, and 3 (5.5%) had subependymal recurrence [31]. The PTV2 they used was similar to the EORTC target volume definition, while PTV1 was smaller than the ESTRO/EANO target volume definition.

Liu et al. retrospectively analyzed 118 adult patients with diffuse glioma and divided them into two groups based on the target treatment volume. In Plan 1 peritumoral edema visible on T2/FLAIR sequences was included while in Plan 2 peritumoral edema was not included. In both plans, CTV was created by adding a 2 cm margin to the GTV. For IMRT planning, the prescribed dose for the PTV was 60 Gy in 30 fractions for both subgroups. There were 40 (63.5%) GBM-diagnosed patients in the Plan 1 group and 36 (65.5%) in the Plan 2 group. They reported that the treatment target volume did not affect outcomes in adult-type diffuse glioma patients. There was also no difference in radiation toxicity (*p* = 0.388). Among the 90 patients who relapsed, 58 (64.4%) had central relapse, 10 (11.1%) had in-field relapse, 3 (3.3%) had marginal relapse, 11 (12.2%) had distant relapse, and 8 (8.9%) had cerebrospinal fluid relapse. According to treatment plans, recurrence patterns were similar, and there was no significant difference in survival [33]. One of the volumes in this study conformed to the RTOG phase I definition, while the other conformed to the EORTC target volume definition. In our study, the mean brain-PTV dose was greater in RTOG volume-based treatment plans than in the EORTC definition, and normal tissues received higher doses. Liu et al. observed no difference between groups in terms of toxicity.

Minniti et al. investigated the recurrence patterns of GBM patients treated with standard chemoradiotherapy using different treatment target volumes. Between 2015 and 2018, 238 patients were treated with IMRT or VMAT using the ESTRO/ACROP target definition receiving 60 Gy in 30 fractions of RT in a single phase [12]. In this study, MRI images of 207 patients with recurrence were fused with simulation CT in the treatment planning system. For each patient, a theoretical plan was created using a 1 cm GTV-to-CTV margin expansion (reduced-CTV plan) to compare failure patterns and radiation doses to the normal brain. When recurrence patterns were compared in the original plan and theoretical plan, intra-field recurrences were 180 and 177, *p* = 0.29; marginal recurrences were 5 and 3, *p* = 0.24; distant recurrences were 22 and 27, *p* = 0.09, respectively [34]. In the same year, 2023, ESTRO/EANO published updated consensus guidelines recommending a single CTV encompassing the resection margin and residual T1-enhancing regions with a 15 mm isotropic margin. They also recommended including FLAIR abnormalities suspected to represent non-enhancing tumors [11].

Qiu et al. included 245 patients with grade 3–4 gliomas treated with standard therapy in their randomized controlled trial evaluating toxicity according to RTOG/NRG (*n* = 122) and EORTC (*n* = 123) target volume definitions. All patients received 60 Gy in 30 fractions of RT using VMAT according to the protocol. There were 59 (48.4%) and 69 (56.1%) patients diagnosed with GBM in the RTOG/NRG and EORTC groups, respectively. No significant difference in neurological toxicity was observed between the RTOG/NRG and EORTC groups. There was no statistical difference in PFS and OS between the two groups. The recurrences observed in the RTOG/NRG and EORTC groups were central 68 (77.3%) and 57 (77%), in-field 4 (4.5%) and 4 (5.4%), marginal 5 (5.7%) and 5 (6.8%), and distant 11 (12.5%) and 8 (10.8%), respectively, and no statistically significant difference was found between the groups [35]. The researchers reported that the limitations of their study included the lack of data on patients’ neurocognitive function, as many patients were unable to complete high-quality neurocognitive assessments during follow-up. In our study, the median initial RTOG/NRG volume was 1.74 times larger than the EORTC volume. All dosimetric parameters were statistically significantly lower in the EORTC volume definition. In the study by Qui et al., this difference was not reflected in either the recurrence pattern, outcomes, or toxicity.

Currently, the use of hippocampus-sparing approaches in radiotherapy of glioblastoma is considered. Bilateral dose sparing of uninvolved hippocampi has been reported to be safe in a large cohort study [36]. In a small prospective observational study of 18 adult patients with benign or low-grade brain tumors treated with conventional fractionated stereotactic radiotherapy, Gondi and colleagues generated a dose–response model showing that a dose equivalent to 2 Gy per fraction greater than 7.3 Gy to 40% of the bilateral hippocampal volume was associated with long-term memory impairment compared to baseline on formal neurocognitive testing at 18 months follow-up [37]. Although the ESTRO/EANO guideline stated that the model was rather imprecise and that the interpretation of 7.3 Gy as a “strict” threshold was not supported, the ESTRO/EANO group reported a consensus that ipsilateral sparing should be discouraged, but considered contralateral hippocampal dose reduction potentially valuable as long as target coverage was maintained (level of agreement: 91%) [11].

Hofmaier and colleagues have shown that using VMAT in glioblastoma radiotherapy can significantly reduce the radiotherapy dose applied to the contralateral hippocampus without compromising other treatment parameters [38].

Fevre et al. included 49 patients in their study examining changes in hippocampal volume according to the dose administered and the location of the glioblastoma. When hippocampi were retrospectively evaluated on three MRI scans taken at baseline, at relapse, and at the end of follow-up, they found that ipsilateral hippocampal volumes were significantly lower than contralateral volumes, regardless of the time of measurement. They also found a significant correlation between the decrease in hippocampal volume and Dmax, D98%, and D40%, regardless of the side. Studies are being conducted to investigate the protection of at least one hippocampus by applying the lowest possible dose to preserve cognitive functions [39].

One of the current research topics is adaptive therapy, which can benefit both normal brain tissue and organs at risk by reducing the dose received. Over the past 15 years, the incorporation of standard and functional MRI sequences into the treatment workflow has become routine, with the increased use of MRI simulators and new integrated MRI-Linac technologies enabling daily pre-, intra-, and post-treatment MRI [40]. Furthermore, the ability to make real-time adaptations throughout the treatment process opens up avenues for identifying biomarkers of response or progression, providing insights into GBM biology and treatment efficacy [41].

A study by the MR-Linac International Consortium Research Group presented contouring recommendations for glioma treatment [42]. Tseng et al. presented the first clinical series of HGG patients treated with RT on an MR-Linac and demonstrated clinically acceptable adaptation-position workflow and treatment times [43].

Guevara et al. demonstrated that weekly adaptive plans on an MR-Linac reduced radiation dose to the hippocampus and brain in the treatment of glioblastoma [44].

A Phase II study, the UNITED study, investigated the feasibility and safety of weekly online adaptive radiotherapy using MR-Linac technology in high-grade gliomas. A reduced CTV margin of 5 mm was used to preserve normal brain tissue while maintaining tumor control. A low marginal risk of relapse (4%) was found without compromising OS or PFS [45]. Studies on adaptive therapy are ongoing.

Our study is the first dosimetric study comparing PTV volumes and OAR doses in treatment plans using the VMAT technique according to five different target definitions in RT of GBM. One of the limitations of our study is that, due to the limited number of patients, it can only present results for a limited number of target volume scenarios based on tumor location and tumor size. Another limitation is that, as a dosimetric study, it cannot provide real-world data on disease recurrence patterns and side effects. However, we believe that the results obtained from comparing different PTV volumes, Brain-PTV Dmean and OAR doses may contribute to the evaluation of real-world data in the literature.

A final limitation we would like to emphasize is that our study was unable to evaluate the target volumes recommended in the ASTRO 2025 guidelines [16]. However, if cone-down/boost is used, the recommendation for a GTV expansion ranging from 10 to 20 mm in each phase, as well as the recommendation for a single-phase volume expansion ranging from 10 to 20 mm in the GTV, makes it impractical to select an ASTRO 2025 volume and compare it with other volume definitions. On the other hand, based on the margins provided by the treatment volume definitions recommended in the ASTRO 2025 guidelines, volumes similar to some of the five different treatment volumes used in our study can be achieved. In this regard, the results of our study can guide the selection of recommendations from the ASTRO 2025 guidelines.

## 5. Conclusions

Various target volume definitions are used in RT for GBM. Volumes that include two phases, peritumoral edema, and wide CTV margins are larger. When these target volume definitions are used, the initial volume is lower than the prescribed total dose, but the irradiated brain tissue volume increases. Keeping OAR doses below dose constraint is mandatory for all volume definitions.

The disease recurrence pattern and observed side effects are important in selecting the target volume definition to be used in treatment. Phase II or retrospective studies have reported no difference in recurrence patterns and side effects between volume definitions using larger or smaller margins. A review of the literature reveals that two-phase volumes with smaller margins and single-phase volumes are more frequently used.

Our findings provide information that the ABTC, EORTC, and ESTRO/EANO protocols may provide advantages in minimizing treatment volumes and OAR doses. Our study demonstrates preliminary dosimetric analysis results that may support the clinical use of the ESTRO/EANO and EORTC protocols with reduced irradiated brain volume, acceptable OAR protection, and practical applicability. In daily practice, clinicians can make their selections by evaluating the radiotherapy device to be used in treatment, treatment planning technique, patient and disease characteristics, recurrence patterns of glioblastoma, limiting doses of organs at risk, and dosimetric studies obtained from different volume definitions.

## Figures and Tables

**Table 1:** Target volume definitions for different groups.

Group	Definition	CTV-Initial	CTV-Boost	PTV
ABTC	Two-phase: 46 + 14 = 60 Gy	T2 + T1E + resection cavity + 5 mm	Resection cavity + T1E + 5 mm	5 mm
EORTC	Single-phase	Resection cavity + T1E + 2 cm	-	5 mm
NCCTG/Alliance	Two-phase: 50 + 10 = 60 Gy	T2 + T1E + resection cavity + 20 mm	Resection cavity + T1E + 20 mm	-
RTOG/NRG	Two-phase: 46 + 14 = 60 Gy	T2 +T1E + resection cavity + 20 mm	Resection cavity + T1E + 20 mm	5 mm
ESTRO/EANO	Single-phase	Resection cavity + T1E + 15 mm (include non-contrast-enhancing tumor if suspected on FLAIR imaging)	-	3 mm

CTV: clinical target volume; T2; T2-weighted MRI sequence; T1E; post-contrast gadolinium-enhanced T1-weighted MRI sequences; ABTC; American Brain Tumor Consortium; NCCTG/Alliance: North Central Cancer Treatment Group/Alliance; RTOG/NRG: Radiation Therapy Oncology Group/NRG; EORTC: European Organization for Research and Treatment of Cancer; ESTRO/EANO: European Society for Radiotherapy & Oncology—European Association of Neuro-Oncology; FLAIR: Fluid attenuation inversion recovery.

**Table 2:** Organ at risk and dose constraints.

Organ at Risk	Dose Constrains	Dose Constraints-Second Criteria
Optic chiasm	Dmax < 54 Gy	Dmax < 60 Gy
Optic nerve	Dmax < 54 Gy	Dmax < 55 Gy
Cochlea	Dmean < 45 Gy	
Brainstem	Dmax < 54 Gy	Dmax < 60 Gy, D59 Gy < 10 cc
Pituitary gland	Dmax < 50 Gy	Dmax < 60 Gy
Eyes	Macula < 45 Gy	
Retina	Dmax < 45 Gy	Dmax < 50 Gy
Lacrimal gland	Dmax < 40 Gy V30 Gy < 50%	
Lens	Dmax < 6 Gy	Dmax < 10 Gy

Dmax: maximum dose; Dmean: mean dose.

**Table 3:** Comparison of data obtained from three initial phase volumes and two single phases.

Parameter	Group A (ABTC) Median (Min–Max)	Group B (NCCTG/Alliance) Median (Min–Max)	Group C (RTOG/NRG) Median (Min–Max)	Group D (EORTC) Median (Min–Max)	Group E (ESTRO/EANO) Median (Min–Max)	*p*-Values
PTV cm^3^	293.8 (100–508.5)	436.4 (213.7–714.6)	519.7 (268–861)	299 (151–694.5)	220.4 (83.8–526.3)	All comparisons *p* < 0.001 * except: A vs. D (*p* = 0.083)
Brain-PTV Dmean Gy	19 (10.2–24.6)	23.9 (15.4–32)	24.6 (16.2–31.3)	24.6 (14.9–37.9)	21.3 (11.8–35.9)	A vs. B, A vs. C, A vs. D (*p* < 0.001 *), A vs. E (*p* = 0.015)

* According to the Bonferroni correction, a *p* value less than 0.005 was considered statistically significant. A: ABTC (American Brain Tumor Consortium); B: NCCTG/Alliance (North Central Cancer Treatment Group/Alliance); C: RTOG/NRG (Radiation Therapy Oncology Group/NRG; D: EORTC (European Organization for Research and Treatment of Cancer); E: ESTRO/EANO (European Society for Radiotherapy & Oncology/European Association of Neuro-Oncology); PTV: planning target volume; Dmean: mean dose; Gy: Gray.

**Table 4:** Comparison of data obtained from three boost volumes, two single phase volumes.

Parameter	Group A (ABTC) Median (Min–Max)	Group B (NCCTG/Alliance) Median (Min–Max)	Group C (RTOG/NRG) Median (Min–Max)	Group D (EORTC) Median (Min–Max)	Group E (ESTRO/EANO) Median (Min–Max)	*p*-Values
PTV cm^3^	144.5 (46–351.3)	233 (109.7–594)	292 (150.6–691)	299 (151–694.5)	220.4 (83.8–526.3)	All comparisons *p* < 0.001 * except: C vs. D (*p* = 0.078)
Brain-PTV Dmean Gy	25.9 (13.5–33.7)	31.5 (19.3–39.8)	34.3 (19.8–41.9)	24.6 (14.9–37.9)	21.3 (11.8–35.9)	All comparisons *p* < 0.001 * except: A vs. D (*p* = 0.481)

* According to the Bonferroni correction, a *p* value less than 0.005 was considered statistically significant. A: ABTC (American Brain Tumor Consortium); B: NCCTG/Alliance (North Central Cancer Treatment Group/Alliance); C: RTOG/NRG (Radiation Therapy Oncology Group/NRG; D: EORTC (European Organization for Research and Treatment of Cancer); E: ESTRO/EANO (European Society for Radiotherapy & Oncology/European Association of Neuro-Oncology); PTV: planning target volume; Dmean: mean dose; Gy: Gray.

## Data Availability

The data presented in this study are available on request from the corresponding author due to ethical reasons.

## Supplementary Materials

The following supporting information can be downloaded at: https://www.mdpi.com/article/10.3390/medicina61101860/s1, Table S1: Comparative analysis of organ-at-risk doses. Table S2. Comparison of data obtained from three initial phase volumes and two single phases and their treatment plans. Table S3. Comparison of data obtained from three boost volumes, two single phase volumes, and treatment plans.

## Abbreviations

The following abbreviations are used in this manuscript:

3D-CRT	Three-dimensional conformal radiation therapy
ABTC	American Brain Tumor Consortium
ASTRO	American Society for Radiation Oncology
CI	Conformity index
CT	Computed tomography
CTV	Clinical target therapy
Dmax	Maximum dose
Dmean	Mean dose
D1cc	Maximum dose to 1 cc
EBRT	External beam radiation therapy
EORTC	European Organisation for Research and Treatment of Cancer
ESTRO	European Organisation for Research and Treatment of Cancer
EANO	European Association of Neuro-Oncology
FLAIR	Fluid attenuation inversion recovery
GBM	Glioblastoma multiforme
GTV	Gross tumor volume
Gy	Gray
HI	Homogeneity index
IMRT	Intensity modulated radiation therapy
MRI	Magnetic resonance imaging
NCCTG	North Central Cancer Treatment Group
OAR	Organ at risk
PTV	Planning target volume
RT	Radiotherapy
RTOG	Radiation Therapy Oncology Group
SD	Standard deviation
VMAT	Volumetric modulated arc therapy
V30	Organ volume receiving >30 Gy
