# Supplementary material for: A Comparative Study of Five Target Volume Definitions for Radiotherapy in Glioblastoma Multiforme
Source: Medicina (Kaunas). 2025 Oct 16;61(10):1860. doi: 10.3390/medicina61101860 (PMC12566177; doi:10.3390/medicina61101860)
Supplement: Supplementary file 1 [file medicina-61-01860-s001.zip › medicina-3901734-supplementary_Table S1.pdf]

Table S1. Comparative analysis of organ-at-risk doses

	A<br>X ± SD<br>Median<br>(min-max)	B<br>X ± SD<br>Median<br>(min-max)	C<br>X ± SD<br>Median<br>(min-max)	D<br>X ± SD<br>Median<br>(min-max)	E<br>X ± SD<br>Median<br>(min-max)	A-B<br>p value	A-C<br>p value	A-D<br>p value	A-E<br>p value	B-C<br>p value	B-D<br>p value	B-E<br>p value	C-D<br>p value	C-E<br>p value	D-E<br>p value
Chiasma<br>Dmax	39±17.2<br>47.9<br>(3.8-53.9)	46±11.7<br>51.6<br>(16.2-53.6)	48.5±9.5<br>52.3<br>(18.5-54.8)	42.3±14.4<br>51<br>(8.5-52.2)	39.4±16.8<br>48.5<br>(4.2-53.7)	**0.002***	**0.001***	0.077	0.861	0.005	0.025	**0.004***	**0.003***	**0.001***	0.014
Cochlea<br>Dmean<br>contralateral	7.6±6.5<br>5<br>(0.6-20.3)	9.6±7<br>6.9<br>(1-22.2)	11±6.6<br>10.7<br>(1.3-20.3)	9.1±6.7<br>7.3<br>(0.9-20.2)	7±6<br>4.4<br>(0.6-20)	0.042	**< 0.001***	0.030	0.352	**0.002***	0.548	0.18	0.006	**0.001***	0.019
Cochlea<br>Dmean<br>İpsilateral	15.4±12.6<br>17.8<br>(0.7-32.3)	17.7±12<br>19.8<br>(1.3-40.4)	20.2±10.9<br>24.6<br>(1.7-33.5)	16.8±11.7<br>20.3<br>(1.2-33.3)	17.4±14.8<br>17.7<br>(0.7-48.2)	0.182	**0.001***	0.161	0.096	0.025	0.360	0.915	**< 0.001***	0.145	0.691
Brainstem<br>Dmax	43.5±17.3<br>51.9<br>(2.3-57.2)	50.4±11.7<br>54.6<br>(5.3-57.5)	52.6±7.6<br>54.9<br>(23.7-58.4)	47.8±11.9<br>52.8<br>(14.7-55.7)	42.8±18.4<br>52.4<br>(4-57)	0.006	**0.004***	0.046	0.570	0.044	0.091	0.008	**0.004***	**0.004***	0.024
Brainstem<br>D1cc	35.6±16.8<br>45<br>(1.8-49.4)	41.8±12<br>48<br>(3.9-51.5)	45.2±9.8<br>48.9<br>(11-52.7)	38.8±14<br>46.7<br>(8.3-49.8)	35.3±17.9<br>44.9<br>(3-53.4)	**0.001***	**0.001***	0.005	0.788	**0.004***	0.011	0.006	**0.001***	**0.001***	0.028
Pituitary<br>gland<br>Dmax	30±16.9<br>33.5<br>(1.8-50)	36.7±14.5<br>43<br>(4.6-51.2)	41±12.1<br>45.1<br>(13.8-51.8)	33.9±16.1<br>38.8<br>(5-49.3)	30.6±17.3<br>33.5<br>(1.6-51)	**< 0.001***	**< 0.001***	0.039	0.636	**< 0.001***	0.041	**0.001***	**< 0.001***	**<0.001***	0.022
Eye Dmax<br>contralateral	16±5.9<br>16<br>(8.6-30.6)	20.6±7.4<br>20<br>(9.1-34)	22.3±8.8<br>20.7<br>(7.9-38.2)	18.1±8<br>16.3<br>(6.8-34.2)	17.7±6<br>17.8<br>(6.8-29)	**< 0.001***	**< 0.001***	0.054	0.021	0.008	0.013	**0.002***	**< 0.001***	**<0.001***	**<0.001***
Eye Dmax<br>ipsilateral	22.7±11.8<br>19<br>(8.6-46.4)	29.8±13<br>30.9<br>(9.8-49)	31.2±13<br>33.2<br>(8.5-49.4)	25.2±14.2<br>20.4<br>(8.9-47.1)	24.2±11.3<br>20.8<br>(7.9-44.4)	**< 0.001***	**< 0.001***	0.112	0.209	0.019	**0.002***	**0.002***	**0.001***	**0.001***	0.422

* According to the Bonferroni correction, a p value less than 0.005 was considered statistically significant.

Abbreviations: A = ABTC (American Brain Tumor Consortium); B = NCCTG/Alliance (North Central Cancer Treatment Group/Alliance); C = RTOG/NRG (Radiation Therapy Oncology Group/NRG; D = EORTC (European Organization for Research and Treatment of Cancer); E = ESTRO/EANO (European Society for Radiotherapy & Oncology – European Association of Neuro-Oncology); SD = Standard deviation; Dmax = Maximum dose; Dmean = Mean dose; D1cc = Maximum dose to 1 cc; V30 = Organ volume receiving >30 Gy.

Table S1. continued (Comparative analysis of organ-at-risk doses)

	A<br>X ± SD<br>Median<br>(min-max)	B<br>X ± SD<br>Median<br>(min-max)	C<br>X ± SD<br>Median<br>(min-max)	D<br>X ± SD<br>Median<br>(min-max)	E<br>X ± SD<br>Median<br>(min-max)	A-B<br>p value	A-C<br>p value	A-D<br>p value	A-E<br>p value	B-C<br>p value	B-D<br>p value	B-E<br>p value	C-D<br>p value	C-E<br>p value	D-E<br>p value
Retina Dmax contralateral	16.2±6.5<br>15.9<br>(8.7-35)	20.9±8.2<br>19.9<br>(9.2-40.9)	22.4±9.3<br>20.5<br>(7.9-43.2)	18.4±8.6<br>16.2<br>(6.8-38)	17.9±6.9<br>17<br>(6.8-35)	**< 0.001***	**< 0.001***	0.05	0.018	0.009	0.012	**0.002***	**< 0.001***	**<0.001***	0.497
Retina Dmax ipsilateral	22.6±11.5<br>19.2<br>(8.5-45.5)	29.2±12.3<br>30.1<br>(9.7-47)	32.2±15.1<br>32.3<br>(8.7-38.7)	24.7±13.7<br>20.3<br>(8.9-46.2)	22.7±9.8<br>20.3<br>(7.7-39.7)	0.163	**< 0.001***	0.165	0.913	0.009	0.012	**0.001***	**< 0.001***	**0.002***	0.184
Lacrimal gland Dmax contralateral	15.6±6.5<br>14.9<br>(6.2-31.2)	20.2±7.6<br>20.4<br>(9.9-34.4)	21.2±7.8<br>22<br>(10-35.5)	18±8.1<br>16.4<br>(6.8-34.7)	17.5±7.2<br>16.8<br>(5.2-30.7)	**< 0.001***	**< 0.001***	0.044	0.019	0.084	0.020	**0.004***	**< 0.001***	**<0.001***	0.480
Lacrimal gland V30 contralateral %	0.04±0.16<br>0<br>(0.7-0.7)	2.42±9.6<br>0<br>(43.1-48.4)	5.7±17.2<br>0<br>(0-74.3)	1.7±6.2<br>0<br>(0-27.6)	0.03±0.13<br>0<br>(0-0.6)	0.282	0.157	0.250	0.330	0.101	0.782	0.281	0.296	0.182	0.249
Lacrimal gland Dmax ipsilateral	22.5±10.6<br>20.6<br>(8.6-37.7)	28.9±10.8<br>31<br>(11.6-43.6)	29.9±10.7<br>32.9<br>(9.8-44)	25.1±12<br>23.4<br>(10.3-40.5)	24.1±10.4<br>25<br>(6.5-39.4)	**<0.001***	**<0.001***	0.047	0.103	0.080	**0.001***	**0.001***	**0.001***	**0.001***	0.291
Lacrimal gland V30 ipsilateral %	6.1±13<br>0<br>(0-46.1)	20.8±26.5<br>1.1<br>(0-81.6)	29.1±37.8<br>7.75<br>(0-99.9)	16.67±24.2<br>0<br>(0-87.4)	15±27<br>0<br>(0-96.1)	0.003*	0.066	0.008	0.089	0.066	0.154	0.076	0.025	0.021	0.676
Lens Dmax contralateral	5.6±1.2<br>5.9<br>(2-7.5)	6.6±1.4<br>6.5<br>(4.4-9.6)	7.6±1.9<br>7.5<br>(4.2-11.8)	5.9±1.7<br>5.6<br>(4.1-10.5)	5.2±1.2<br>5.2<br>(1.6-7.5)	**<0.001***	**<0.001***	0.295	0.017	**<0.001***	0.005	**<0.001***	**<0.001***	**<0.001***	0.005
Lens Dmax ipsilateral	6.2±1.98<br>6.2<br>(1.9-10.5)	7.4±2.3<br>7.3<br>(4.1-12.2)	8.2±2.6<br>7.9<br>(4.4-13)	6.7±2.6<br>6.2<br>(3.1-12.4)	5.5±1.7<br>5.1<br>(1.6-9.2)	**<0.001***	**<0.001***	0.101	0.006	0.001*	0.020	**<0.001***	**<0.001***	**<0.001***	**<0.001***

* According to the Bonferroni correction, a p value less than 0.005 was considered statistically significant.

Abbreviations: A = ABTC (American Brain Tumor Consortium); B = NCCTG/Alliance (North Central Cancer Treatment Group/Alliance); C = RTOG/NRG (Radiation Therapy Oncology Group/NRG; D = EORTC (European Organization for Research and Treatment of Cancer); E = ESTRO/EANO (European Society for Radiotherapy & Oncology – European Association of Neuro-Oncology); SD = Standard deviation; Dmax = Maximum dose; Dmean = Mean dose; D1cc = Maximum dose to 1 cc; V30 = Organ volume receiving >30 Gy.
