# Supplementary material for: A Comparative Study of Five Target Volume Definitions for Radiotherapy in Glioblastoma Multiforme
Source: Medicina (Kaunas). 2025 Oct 16;61(10):1860. doi: 10.3390/medicina61101860 (PMC12566177; doi:10.3390/medicina61101860)
Supplement: Supplementary file 1 [file medicina-61-01860-s001.zip › medicina-3901734-supplementary_Table S2.pdf]

Table S2. Comparison of data obtained from three initial phase volumes and two single phases and their treatment plans.

Parameter	Group A (ABTC) X ± SD Median (min-max)	Group B (NCCTG/Alliance) X ± SD Median (min-max)	Group C (RTOG/NRG) X ± SD Median (min-max)	Group D (EORTC) X ± SD Median (min-max)	Group E (ESTRO/EANO) X ± SD Median (min-max)	Significant p-values
PTV cm^3	295.2±98.7 293.8 (100–508.5)	438.7±130.3 436.4 (213.7–714.6)	517.2±143.7 519.7 (268–861)	329.4±132 299 (151–694.5)	226.9±105.2 220.4 (83.8–526.3)	A vs B, A vs C, A vs E, B vs C, B vs D, B vs E, C vs D, C vs E, D vs E (all p<0.001*), A vs D (p=0.083)
Brain-PTV Dmean Gy	18.9±3.8 19 (10.2–24.6)	24.8±4.3 23.9 (15.4–32)	24.2±3.8 24.6 (16.2–31.3)	25±5.2 24.6 (14.9–37.9)	21.6±5.6 21.3 (11.8–35.9)	A vs B, A vs C, A vs D (p<0.001*), A vs E (p=0.015), B vs E (p=0.007), C vs E (p=0.006), D vs E (p<0.001*)
HI	0.06±0.02 0.05 (0.04–0.10)	0.06±0.02 0.07 (0.04–0.11)	0.08±0.03 0.07 (0.04–0.14)	0.06±0.02 0.05 (0.04–0.11)	0.03±0.2 0.03 (0.01–0.09)	–
CI	1.05±0.03 1.05 (1.02–1.14)	1.04±0.02 1.04 (1.01–1.08)	1.03±0.02 1.03 (1.00–1.09)	1.04±0.02 1.04 (1.00–1.07)	1.00±0.01 1.00 (1.00–1.02)	–

*According to the Bonferroni correction, a p value less than 0.005 was considered statistically significant.

A: ABTC (American Brain Tumor Consortium); B: NCCTG/Alliance (North Central Cancer Treatment Group/Alliance); C: RTOG/NRG (Radiation Therapy Oncology Group/NRG; D: EORTC (European Organization for Research and Treatment of Cancer); E: ESTRO/EANO (European Society for Radiotherapy & Oncology/European Association of Neuro-Oncology); PTV: planning target volume; Dmean: mean dose; Gy: Gray; HI = homogeneity index; CI: conformity index.
