# Supplementary material for: A Comparative Study of Five Target Volume Definitions for Radiotherapy in Glioblastoma Multiforme
Source: Medicina (Kaunas). 2025 Oct 16;61(10):1860. doi: 10.3390/medicina61101860 (PMC12566177; doi:10.3390/medicina61101860)
Supplement: Supplementary file 1 [file medicina-61-01860-s001.zip › medicina-3901734-supplementary_Table S3.pdf]

Table S3. Comparison of data obtained from three boost volumes, two single phase volumes, and treatment plans.

Parameter	Group A (ABTC) X ± SD Median (min-max)	Group B (NCCTG/Alliance) X ± SD Median (min-max)	Group C (RTOG/NRG) X ± SD Median (min-max)	Group D (EORTC) X ± SD Median (min-max)	Group E (ESTRO/EANO) X ± SD Median (min-max)	Significant p-values
PTV cm³	156.8±72<br>144.5<br>(46–351.3)	256.8±114.5<br>233<br>(109.7–594)	322.5±130<br>292<br>(150.6–691)	329.4±132<br>299<br>(151–694.5)	226.9±105.2<br>220.4<br>(83.8–526.3)	A vs B, A vs C, A vs D, A vs E, B vs C, B vs D, B vs E, C vs E, D vs E (all p<0.001*), C vs D (p=0.078)
Brain-PTV Dmean Gy	25.7±5.2<br>25.9<br>(13.5–33.7)	32±5.4<br>31.5<br>(19.3–39.8)	34±5.6<br>34.3<br>(19.8–41.9)	25±5.2<br>24.6<br>(14.9–37.9)	21.6±5.6<br>21.3<br>(11.8–35.9)	All comparisons p<0.001* except: A vs D (p=0.481), A vs E (p=0.002*)
HI	0.03±0.02<br>0.04<br>(0.03–0.08)	0.05±0.01<br>0.05<br>(0.03–0.09)	0.05±0.02<br>0.04<br>(0.03–0.08)	0.06±0.02<br>0.05<br>(0.04–0.11)	0.03±0.2<br>0.03<br>(0.01–0.09)	–
CI	1.21±0.08<br>1.22<br>(1.11–1.47)	1.25±0.08<br>1.25<br>(1.13–1.52)	1.18±0.05<br>1.17<br>(1.11–1.32)	1.04±0.02<br>1.04<br>(1.00–1.07)	1.00±0.01<br>1.00<br>(1.00–1.02)	–

*According to the Bonferroni correction, a p value less than 0.005 was considered statistically significant.

A: ABTC (American Brain Tumor Consortium); B: NCCTG/Alliance (North Central Cancer Treatment Group/ Alliance); C: RTOG/NRG (Radiation Therapy Oncology Group/NRG; D: EORTC (European Organization for Research and Treatment of Cancer); E: ESTRO/EANO (European Society for Radiotherapy & Oncology/European Association of Neuro-Oncology); PTV: planning target volume; Dmean: mean dose; Gy: Gray; HI: homogeneity index; CI: conformity index.
